# Photosynthesis, Nitrogen Allocation, Non-Structural Carbohydrate Allocation, and C:N:P Stoichiometry of *Ulmus elongata* Seedlings Exposed to Different Light Intensities

**DOI:** 10.3390/life12091310

**Published:** 2022-08-25

**Authors:** Guangyu Luo, Junmin Li, Shuiliang Guo, Yueling Li, Zexin Jin

**Affiliations:** 1 College of Life Sciences, Shanghai Normal University, Shanghai 200234, China; 2 Institute of Ecology, Taizhou University, Taizhou 318000, China; 3 Zhejiang Provincial Key Laboratory of Plant Evolutionary Ecology and Conservation, Taizhou University, Taizhou 318000, China

**Keywords:** photosynthetic parameters, C:N:P stoichiometry, nitrogen allocation, *Ulmus elongata*, non-structural carbohydrates, rare and endangered plants

## Abstract

The leaf photosynthetic capacity, leaf N partitioning, non-structural carbohydrate content, C, N, and P contents of endangered *U. elongata* seedlings exposed to different light intensities were compared in this study. The most favorable light condition for the survival and growth of *U. elongata* seedlings in the present study was 100% full sunlight, as this induced higher *P*_n_, PNUE, *P*_C_, *P*_R_, *P*_B_, and NSC content relative to shade-treated seedlings. PNUE, *P*_R_, *P*_C_, and *P*_B_ in *U. elongata* seedling leaves decreased under 40% and 10% full sunlight, while *P*_L_ increased, indicating that shade increased the light capture efficiency of photosystem (PS) II but decreased electron transfer from PSII to PSI. Furthermore, leaf N content increased with shade intensity, revealing an adaptive strategy for poor light environments. Additionally, the smallest leaf biomass, *P*_n_, WUE, and *CE* values and C:N and C:P ratios in stems and leaves were observed under 10% full sunlight. These results indicate that seedlings growing under 40% full sunlight will benefit *U*. *elongata* conservation.

## 1. Introduction

Light is a critical abiotic factor affecting the survival, growth, and development of plant [1]. Changes in light induce various physiological responses in plants [2], which have evolved complex physiological adaptations that allow them to withstand combinations of stresses imposed by unfavorable light levels [3]. Exploring the adaptive responses of plants to various light environments can help to elucidate the breadth of their environmental niches. 

Low light decreases the photosynthesis and consequently reduces carbon accumulation and growth of a plant. The carbon gain hypothesis, which posits that seedlings of plants tend to maximize net carbon gain to perform optimally in low-light conditions [4,5], suggests that shade-tolerant species should increase investments of N to compounds capable of light capture (e.g., chlorophyll and other pigments) [6,7,8] and alter plant morphology to maximize light capture [9,10]. In addition, to effectively balance N allocation, plant may invest more N in light harvesting proteins to maximize the carbon gain in low-light conditions, whereas in RuBisCO and bioenergetic components to maximize carbon gain in high-light conditions [11,12]. When the carbon gain is greater than the carbon demand for growth, plants deposit photosynthates as nonstructural carbohydrate (NSC), which can be mobilized later to support the survival, growth, or other functions [4,13,14], such as the tolerance to shade [15,16].

Variation in the C, N, and P contents of plant tissues are owed to the balance between absorbance and storage [17]. The stoichiometry of leaves is more sensitive to shade treatments than that of roots because leaves are the major photosynthetic organ [18]. The C:N:P stoichiometry is related to plant survival and growth strategies, such as an organism’s capability of adapting to environmental stresses [19]. Shade treatments differentially affect the C:N:P stoichiometry of plant organs [18,20]. However, the specific mechanisms that induce changes in plant stoichiometry at the plant organ level in response to light intensity changes are understudied.

*Ulmus elongata*, a unique rare species with small populations in China [21], has been listed in the IUCN Red List of Endangered Species (Vulnerable VU level) and the first batch of National Key Protected Wild Plants (list II level) in China [22,23,24]. Most previous studies have focused on the conservation value and endangered status of *U. elongata*. The eco-physiological response of *U. elongata* to light with different intensities has not been studied [25]. Previous studies showed that *U. elongata* seedlings rarely establish under canopies and that its seeds are dispersed only over short distances, exacerbating its current conservation status [26,27]. Thus, assessment of the effect of shading on the physiology, especially the photosynthesis process of *U. elongata*, can be used to identify appropriate light intensities for *U. elongata* seedling establishment and thus inform *U. elongata* conservation efforts.

Here, the leaf photosynthetic capacity, leaf N partitioning, NSCs, C, N, and P contents, and C:N:P stoichiometry of *U. elongata* seedlings under different light intensity levels were measured. We aimed to determine the responses of *U*. *elongata* leaves under different light intensities with respect to (1) photosynthesis, (2) nitrogen allocation, (3) carbohydrate storage allocation, and (4) C:N:P stoichiometry. These results elucidate the optimal light-intensity conditions for *U. elongata* seedlings and provide a basic reference for the conservation of *U. elongata*.

## 2. Materials and Methods

### 2.1. Plant Materials

In 2017, seeds were planted at the Forestry Science Research Institute, Lishui City, Zhejiang Province, China. In 2019, the surviving seedlings were transferred to Taizhou University, Taizhou, Zhejiang Province, China (121°7′ E, 28°51′ N). The pot experiment was established in a greenhouse at Taizhou. Each seedling was transplanted into plastic pots filled with humidified nutrient soil. The 2-year-old seedlings were watered every day to pot water capacity. The soil physio-chemical traits were shown in Table S1.

### 2.2. Treatments

In March 2019, three treatments were established using a black nylon to control the light intensity. Treatments conducted with zero, one, or two layers of shading net correspond to 100%, 40%, and 10% full sunlight, respectively [28].

### 2.3. Photosynthetic Parameter Measurement

In July 2019, three healthy third leaves from the top of each selected *U. elongata* were used for the measurement of photosynthetic characteristics on a clear day. Three individuals from each treatment were randomly selected. The diurnal variation in air temperature (*T*_a_), photosynthetically active radiation (PAR), and relative humidity (RH) under three treatments are shown in Figure S1. The photosynthetic parameters were measured with the LI-COR 6400 system (LI-COR 6400, Lincoln, NE, USA) at 1000 μmol·m^−2^·s^−1^ PAR, 400 μmol·mol^−1^ CO_2_, 60% relative humidity, and the leaf temperature was kept at 25 °C. The photosynthetic rate (*P*_n_), intercellular CO_2_ concentration (*C*_i_), stomatal conductance (*G*_s_), and transpiration rate (*T*_r_) of *U. elongata* were determined from 9:00 to 11:00 on a clear day. Water use efficiency (WUE) was calculated as *P*_n_*/T*_r_ [29,30]. Carboxylation efficiency (*CE*) was the ratio of *P*_n_ to *C*_i_, and the stomatal limitation (*L*_s_) value was calculated as 1-(*C*_i_/*C*_a_) (here, *C*_a_ is the atmospheric CO_2_ concentration) [31,32]. 

The *P*_n_—PAR curves were logged under PAR of 0, 20, 50, 100, 150, 400, 600, 800, 1000, 1200, 1500, and 2000 μmol·m^−2^·s^−1^ levels, using LED blue and red light sources [28]. The CO_2_ response curves were measured under saturated PAR. The parameters were measured at 500 μmol·s^−1^ gas flow rate, 60% RH, and the leaf temperature was kept at 25 °C. The *P*_n_—*C*_i_ curves were logged under *C*_i_ of 50, 100, 200, 300, 400, 600, 800, 1000, 1200, and 1500 μmol·mol^−1^ levels. The maximum electron transport rate (*J*_max_) and the maximum carboxylation rate (*V*_cmax_) were calculated by the method of Long and Bernacchi [33]:

*V*_cmax_ = *k* × [*C*_i_ + *K*_c_ × (1 + *O*/*Ko*)]^2^/[*Γ* * + *K*_c_ × (1 + *O*/*Ko*)],

*J*_max_ = [4 × (*A*_max_ + *R*_d_) × (*C*_i_ + 2*Γ* *)]/[*C*_i_ – *Γ* *].

Here, *C*_i_ is the intercellular CO_2_ concentration, and *O* is the intercellular O_2_ concentration; *Γ* * is the CO_2_ compensation point; *Ko* and *K*c are Michaelis–Menten constants for oxygenation and carboxylation, respectively; *R*_d_ is the dark respiration rate, and *A*_max_ is the light-saturated photosynthetic rate [28,33].

### 2.4. N Allocation

After the determination of the photosynthetic parameters, 20–30 leaves from each *U. elongata* seedling were selected. Leaf area (A, cm^2^) was measured using a WinFOLIA multipurpose area meter (Regent Instruments Inc., Sainte-Foy, Quebec, QC, Canada). Leaf biomass was measured after being oven-dried at 80 °C. *M*_A_ (g·m^−2^) was the ratio of leaf biomass to leaf area [34]. Three replicates were used for every treatment.

Subsequently, dried leaves of *U. elongata* were ground into powder and the leaf N per unit mass (*N*_mass_, g·g^−1^) was measured by ICP-OES (Optima 2100DV, PerkinElmer) after H_2_SO_4_-H_2_O_2_ solution digestion and dilution [35]. The leaf N per unit area (*N*_area_, g·m^−2^) value was then determined as *N*_mass_ × *M*_A_, while the PNUE (μmol·g^−1^·s^−1^) was the ratio of *P*_n_ to *N*_area_ [36,37,38].

Fractions of leaf N allocated to RuBisCO (*P*_R_), bioenergetics (*P*_B_), light-harvesting (*P*_L_), and photosynthetic (*P*_C_) components were calculated using the following equation:

*P*_R_ = *V*_cmax_/(6.25 × *V*_cr_ × *M*_A_ × *N*_mass_),

*P*_B_ = *J*_max_/(8.06 × *J*_mc_ × *M*_A_ × *N*_mass_),

*P*_L_ = *C*_Chl_/(*N*_mass_ × *C*_B_),

*P*_C_ = *P*_R_ + *P*_B_ + *P*_L_.

Here, *N*_mass_ is the leaf N content per unit of leaf dry mass (g·g^−1^); *M*_A_ is leaf dry mass per unit area (g·m^−2^); *C*_Chl_ is leaf chlorophyll content (mmol·g^−1^); *V*_cr_ and *J*_mc_ are the specific activity of RuBisCO and the potential rate of photosynthetic electron transport per unit cytochrome f, respectively; and *C*_B_ is the ratio of leaf chlorophyll to leaf nitrogen invested [39,40,41,42]. *C*_B_, *V*_cr,_ and *J*_mc_ were calculated using the following equation:

[*C*_B_] = 1.94 + 12.6/[*M*_A_],

$$V_{\mathrm{cr}}\left(J_{\mathrm{mc}}\right)={\;e}^{\left(c-\frac{\Delta H_{a}}{R\times T_{k}}\right)}/\left(1+e^{\frac{\Delta S\times T_{k}-\Delta H_{d}}{R\times T_{k}}}\right).$$

Here, [*M*_A_] and [*C*_B_] are the values of *M*_A_ and *C*_B_, respectively; *M*_A_ is the leaf dry mass per unit area (g·m^−2^); *C*_B_ is the ratio of leaf chlorophyll to leaf nitrogen invested in light harvesting; and *R* is the gas constant (8.314 J·K^−1^·mol^−1^), ∆*S* is the entropy term, c is the scaling constant, ∆*H*a is the activation energy, *T*_k_ is the leaf temperature (K), and *∆H*_d_ is the deactivation energy [36,37,39,43].

### 2.5. Determination of C, N, and P Contents

The biomass of roots, stems, and leaves of *U. elongata* were measured after they were kept at 80 °C. The dry root, stem, and leaf samples of *U. elongata* were also ground into powder with a Mixer Mill grinder (High speed multifunctional grinder, 4000 r/min, Yongkang Baoou Hardware Products Co., Yongkang, China). These powdered samples were passed through a screen with a 1-mm mesh. The C content was determined by the H_2_SO_4_/K_2_Cr_2_O_7_ oxidization-FeSO_4_ titration method [35,44]. After H_2_SO_4_-H_2_O_2_ solution digestion and dilution, total N and P content were determined by ICP-OES (Optima 2100DV, PerkinElmer) [35]. The N:P, C:P, and C:N ratios were calculated from the content ratio [45]. Three replicates were used for every treatment.

### 2.6. Determination of NSC Content

To determine the NSC content, 5 mL of distilled water was added into 50 mg of the powdered samples of *U. elongata* and the mixture was incubated for 45 min in a boiling water bath. The supernatant was collected. The process was performed twice to ensure complete extraction of all sugars [46]. The supernatant was dried and resolved in 10 mL of 30% (*v*/*v*) perchlorate. Soluble sugar and starch contents were determined at 630 nm using the anthrone colorimetric method according to a glucose standard curve. NSC content was calculated as the sum of soluble sugar and starch content [45,47]. Three replicates were used for every treatment. Soluble sugar-to-starch ratio (SSRs) was calculated as the soluble sugar content divided starch content. 

### 2.7. Statistical Analyses

Data were analyzed with SPSS version 20.0 (IBM Corp., Armonk, NY, USA). We used a one-way analysis of variance with treatment as the fixed factor to test the significant difference of the photosynthetic parameters, PNUE, *P*_R_, *P*_B_, *P*_L_, *P*_C_, C, N, and P contents, N:P, C:P, C:N, sugar, starch, NSC, and SSRs. One-way ANOVA with the least significant difference (LSD) test was applied to detect significant differences at a *p* < 0.05 level. Figures were drawn with Origin 8.5 (Origin Lab., Northampton, MA, USA). 

## 3. Results

### 3.1. Leaf Photosynthetic Capacity and Biomass Response to Light Intensity Levels

**Figure 1:**
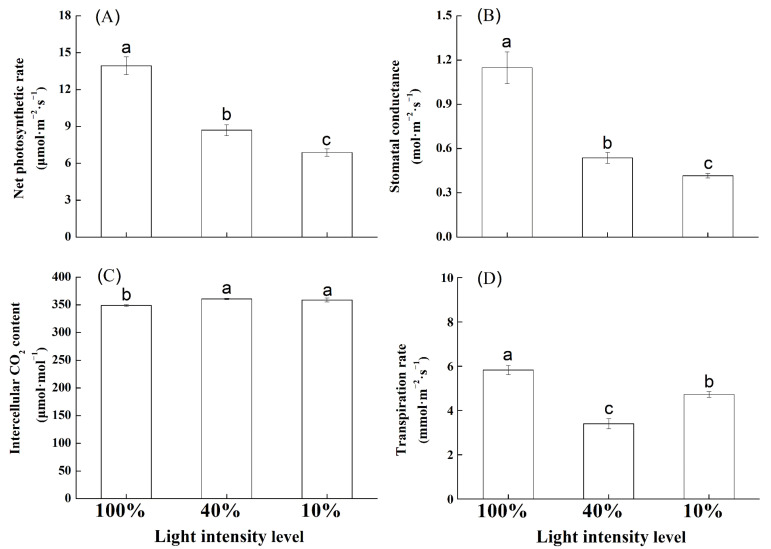
Photosynthetic parameters in *Ulmus elongata* leaves under different light intensity levels. (**A**) Net photosynthetic rate (*P*_n_); (**B**) Stomatal conductance (*G*_s_); (**C**) Intercellular CO_2_ concentration (*C*_i_); (**D**) Transpiration rate (*T*_r_). Data are presented as the mean ± SE. For each panel, bars labeled with different small letters indicate a significant difference at 0.05 levels.

The photosynthetic parameters of *U. elongata* leaves changed significantly under different light intensities (Figure 1). As light intensity decreased, *P*_n_ decreased continuously with maximum and minimum values under 100% and 10% full sunlight, respectively (Figure 1A). The differences among treatments were significant (*p* < 0.05). The trend in *G*_s_ was similar to that of *P*_n_ (*p* < 0.05) (Figure 1B). *C*_i_ reached its minimum under 100% full sunlight, which was significantly different from those at 40% and 10% full sunlight (*p* < 0.05) (Figure 1C). Differences in *T*_r_ among the three treatments were significant (Figure 1D). *L*_s_ and *T*_r_ were at their lowest under 40% full sunlight, while WUE under 40% full sunlight was at its highest (*p* < 0.05; Table 1). *CE* decreased significantly with the decreasing of light intensity (Table 1). In addition, leaf biomass at 10% full sunlight was lower than that at 40% and 100% full sunlight (Figure S2).

### 3.2. N Allocation Responses to Light Intensity Levels

The fraction of leaf N allocated to photosynthetic components differed among light intensity levels (Table 2). *P*_L_ increased with the decreasing of light intensity levels. *P*_R_ and *P*_B_ decreased as the light intensity decreased (*p* < 0.05), which indicates that the shade increased the light capture efficiency of PSII in *U. elongata* leaves. PNUE and *P*_C_ were reduced as the light level decreased.

### 3.3. C, N, and P Contents and C:N:P Stoichiometry Response to Light Intensity Levels

**Figure 2:**
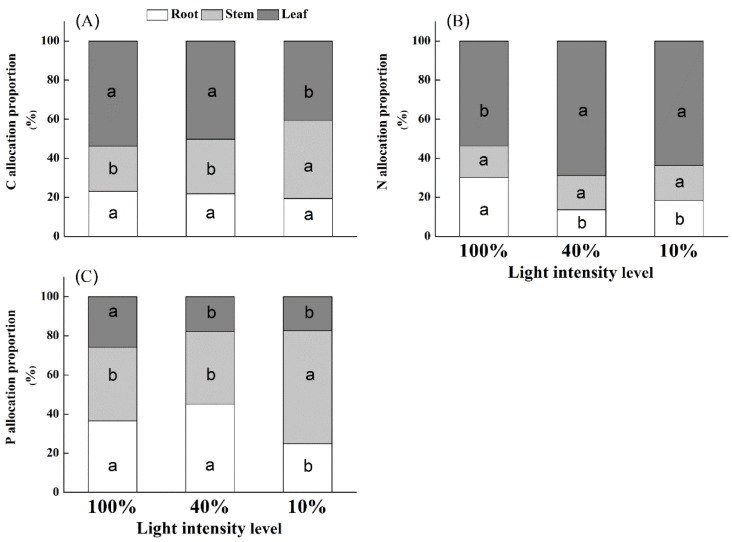
Allocation proportion of carbon (**A**), nitrogen (**B**), and phosphorus (**C**) in the leaf, stem, and root tissues of *Ulmus elongata* seedlings in response to light intensity levels. For each panel, bars labeled with different letters indicate a significant difference at 0.05 levels.

Shade treatments significantly affected the allocation proportion of carbon, phosphorus, and nitrogen in the leaf, stem, and root tissues of *U. elongata* (Table 3; Figure 2). The leaf and root C contents were reduced as the light intensity decreased, while the stem C content increased as the light level decreased (Table 3). The leaf N content in *U.* *elongata* seedlings significantly increased from 100% to 10% full sunlight (*p* < 0.05) (Table 3). The highest stem N content was observed under 10% full sunlight. Leaf P content in *U. elongata* seedlings significantly decreased from 100% to 10% full sunlight, and the lowest root P content was observed under 10% full sunlight (Table 3). In contrast, the stem P content in *U. elongata* seedlings reached its maximum under 10% full sunlight (*p* < 0.05).

Shade treatments significantly affected the root, stem, and leaf C:N:P stoichiometries of *U. elongata* (Table 4). The C:N ratio in roots was highest under 40% full sunlight and was significantly different from that under 100% and 10% full sunlight, while the C:P and N:P ratios were highest under 10% full sunlight (Table 4). In *U. elongata* seedlings, the stem and leaf N:P ratios increased as the light intensity decreased. In contrast, the stem and leaf C:N ratios decreased as the light level decreased. The stem and leaf C:P ratios of *U. elongata* were highest under 40% full sunlight. These results indicated that the lowest N- and P-use efficiency and growth rate occurred under the deep shade treatment.

### 3.4. NSC Content in Response to Light Intensity Levels

**Figure 3:**
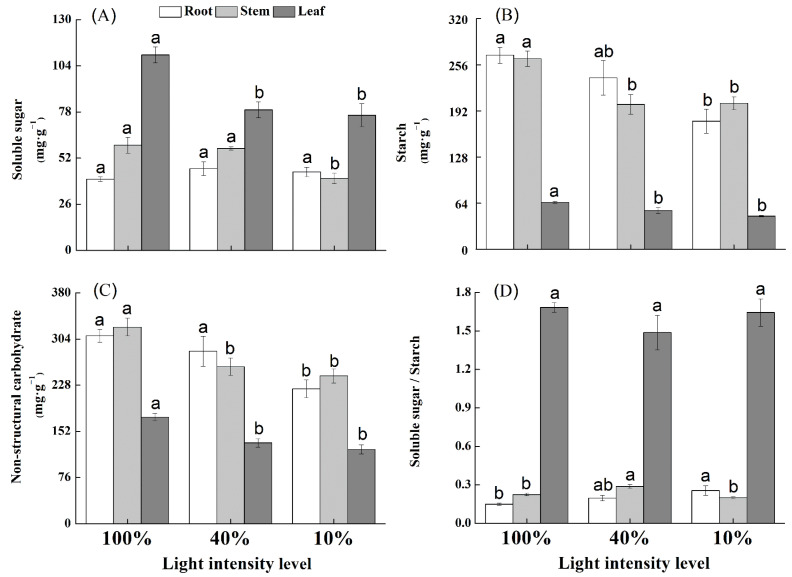
Soluble sugar content (**A**), starch content (**B**), non-structural carbon (NSC) content (**C**), and soluble sugar-to-starch ratio (SSRs) (**D**) in leaf, stem, and root tissues of *Ulmus elongata* seedlings in response to light intensity levels. For each panel, bars labeled with different letters indicate a significant difference at 0.05 levels.

The shade treatments had significant effects on leaf soluble sugar, starch, and NSC contents of *U. elongata* seedlings (Figure 3). The soluble sugar content in stem tissue was at its lowest under 10% full sunlight and significantly different from that under 100% and 40% full sunlight (Figure 3A). The root sugar content was similar among different light intensities, but the root starch content decreased significantly as the light level decreased from 100% to 40% full sunlight (Figure 3B). Starch and NSC contents in stem tissue under 100% full light were higher than shade treatments (40% and 10% full sunlight) (*p* < 0.05; Figure 3C). 

In *U. elongata*, shade treatments significantly affected the root SSRs. The SSRs increased as the light level decreased (Figure 3D), indicating that soluble sugar is preferentially used by roots. Accordingly, the roots began to utilize the available starch under low light intensity (40% and 10% full sunlight). In *U. elongata*, light levels had a limited effect on stem SSRs, which peaked under 40% full sunlight and was relatively similar under the other light levels. This study showed no significant difference in leaf SSRs of *U. elongata* seedlings among the different light levels (*p* > 0.05).

## 4. Discussion

Light is a critical factor providing energy to photosynthesis [48]. However, plants evolve complex adaptation strategies to cope with light stress [28]. In our study, *U. elongata* seedlings grown under 100% sunlight had higher *P*_n_ than those seedlings grown under either of the shading treatments. The decrease in *P*_n_ in response to shade was consistent with the observed *G*_S_ decrease. In contrast, *C*_i_ increased or remained unchanged under shade conditions. These results suggested that the decrease in *P*_n_ was related to nonstomatal limitations [49]. The significant decreases of *P*_n_ and *G*_S_ in *U. elongata* seedlings under shading treatments might be owed to the inhibition of carboxylation efficiency, and/or reallocation of leaf nitrogen [11,12]. Notably, the carboxylation efficiency (*CE*) was reduced under both 40% and 10% full sunlight, which strongly suggests that the decrease in photosynthesis was caused by nonstomatal factors. These findings indicate that a light intensity less than that of 40% full sunlight imposes light limitation, resulting in a reduction in the photosynthetic enzymatic activity of *U. elongata* seedlings. On the other hand, the smallest leaf biomass, *P*_n_, WUE, and *CE* values under 10% full sunlight indicated that 10% full sunlight led to a rapid decline in plant growth and development. Excessive shade has become the critical limiting factor for *U. elongata* seedling photosynthetic capacity. Similar results were indicated in endangered *Tetrastigma hemsleyanum* [29].

Relevant studies on N allocation have revealed that plants under low light invest more N in light harvesting proteins to maximize carbon gain, as was observed in *Juglans nigra*, *Panax notoginseng*, and *Abies alba* [6,11,42,50,51]. In contrast, high investment of N in RuBisCO and bioenergetic components, which increases the photosynthetic capacity under high light, was observed in *Prunus persica* and *Picea abies* [7,50,51]. Plants under shade conditions allocate more N to *P*_L_ to capture more light energy, and less N to *P*_B_, *P*_R_, and *P*_C_. This N allocation possibly caused a time-lag response of carboxylation [42]. The allocation of leaf N to proteins, for example, PNUE and *P*_n_, were affected by light intensity levels [11,50]. In the present study, PNUE, *P*_R_, *P*_C,_ and *P*_B_ decreased, and *P*_L_ increased under low-light (i.e., 40% and 10% full sunlight) in the leaves of *U. elongata* seedlings. These results indicated that shade increased the light capture efficiency of PSII in *U. elongata* leaves but decreased the capacity of electron transport. This mechanism might contribute to the observed decreased in photosynthetic capacity under shading of *U. elongata* seedlings.

The leaf N allocation patterns were affected by CO_2_ fixation and light-harvesting proteins [11,12]. When the light availability increased, the plants increased N investment in the electron carriers, Calvin cycle enzymes, RuBisCO, and bioenergetic proteins to compete more pigment compounds, such as carotenoids and flavonoids, and antioxidants to protect leaves against excess light [11,51]. These results indicate that *U. elongata* under shade treatment has evolved to promote the absorbed N, being preferentially transferred from vegetative parts to photosynthetic pigments to obtain an imbalance between C accumulation and N supply [52]. Therefore, the balance between the acquisition and utilization of light energy of *U. elongata* seedlings may be regulated according to light intensity levels. 

N is a critical component of all proteins, such as functional proteins, structural proteins, and photosynthetic proteins. It is necessary for plants to allocate more N to structural proteins for stronger structural defenses under light stress [17,53]. P is the main constitute of ribosomal RNA, which plays an important role in the synthesis of proteins [17]. In *U. elongata*, in contrast to the observed decrease in leaf P content, as light was reduced, an increasing tendency of leaf N revealed adaptive strategies for poor light environments. In contrast to the greater requirements for proteins and nucleic acids under 100% full sunlight condition, *U. elongata* seedlings may use more N resources to synthesize light-trapping proteins under shade treatments [46].

The allocation of nutrients (i.e., C, N, and P) among different plant tissues is critical for plant functional diversity [17,54]. The transport of nutrients caused by changes in the balance between element uptake and utilization efficiency may lead to different C, N, and P contents across plant organs [17,55]. In this study, the C, N, and P contents of *U. elongata* were significantly affected by both light intensity and organ type. Additionally, the variation in nutritional elements (C, N, P) in different organs of *U. elongata* under light stress suggested the existence of strategies in balancing nutritional metabolism and adapting to light stress [17].

In this study, we also found that C and N contents of the leaves of *U. elongata* seedlings were significantly higher than those in the stem and root tissues, indicating that the allocation of nutritional elements to leaves was prioritized compared with the stems and roots [17,56]. The stem C, N, and P contents of *U. elongata* seedlings significantly increased under 10% full sunlight. This can be explained by two distinct mechanisms. First, the total biomass of C, N, and P allocation to the above-ground (especially for stem organs) decreased in *U. elongata* seedlings under 10% full sunlight, which may lead to an increase in nutrient concentration (i.e., enrichment effect) [57]. Different plant size and biomass can cause the variation of nutritional elements in plant tissues according to a dilution effect [14]. The contents of nutritional elements in the stem also decrease with ontogenetic development [17,58]. Second, there was a higher proportion of C-enriched structural compounds in stem issues under 10% full sunlight, such as lignin and cellulose [46,59]. These results indicated that changes in the profiles of different nutrient elements of *U. elongata* seedlings may be related to the nutrient use efficiency under different intensity levels [17].

C:N:P stoichiometry reflects the trade-off between growth and stress tolerance [60]. We found lower C:N and C:P ratios in stem and leaf tissues under 10% full sunlight, indicating that the lowest nutrient use efficiency and growth rate occurred under the deep shade treatment. Variation in the C:N and C:P ratios throughout the growth season can be used to indicate the nutrient use efficiency of plants under different light intensity levels [17]. Thus, the C:N ratio in stem and leaf tissues increased from 10% to 100% full sunlight, indicating that the compounds with high C:N or C:P ratio accumulated in *U. elongata* seedlings under 100% full sunlight.

The leaf N:P ratio can be used to evaluate the limiting patterns of nutrients [52,61]. Generally, ratios of <14 and >16 are interpreted as indicating N and P limitation, respectively [17,62]. In the present study, the leaf N:P ratio of *U. elongata* was <14 under 100% full sunlight, while >16 under shade treatments. Additionally, when the light intensities increased from 10% to 100% full sunlight, the leaf N:P ratio decreased. These results indicate that the growth of *U. elongata* was much more limited by P than by N under shade treatments (i.e., 10% and 40% of full sunlight), which was consistent with the fact that *U. elongata* is a fast-growing plant and has a low N:P ratio under 100% full sunlight [17]. In addition, we found a similar trend in N:P variation in the leaf and stem tissues, which can be used to infer potential N- or P- limitation. This stoichiometric mechanism can clarify plant survival strategies in response to light intensity levels of *U. elongata* seedlings. In this study, higher C, N, and P contents under 100% full sunlight were found in roots, indicating active uptake of these nutrients. Further studies are needed to elucidate the underlying mechanism. 

NSCs are an important energy source and comprise a necessary temporary solute source for transport metabolism and osmoregulation in plant growth and metabolism [63]. NSCs also function as physiological traits corresponding to ecologically adaptive strategies [63,64] and thus have been linked to the capacity to withstand stress such as high light intensity [46,64]. In the present work, we found that the 100% light intensity level resulted in significantly higher NSCs contents in the leaves of *U. elongata* seedlings. Liu et al. (2020) found that seedlings of *Schima superba* under light limitation used their carbon stores for growth [45]. Additionally, our results show that *U. elongata* seedlings tend to preferentially supply more stored NSCs in roots and stems relative to leaves. Increased available NSCs in the roots and stems may be used for root growth and defense against stress conditions such as 100% light intensity level [63,65], as *U. elongata* seedlings can extend their roots to increase their range of water and minerals absorption [65,66]. In the present study, we observed the lowest *P*_n_ and a relatively low total NSC content of *U. elongata* seedlings under 10% full sunlight, which may lead to their poor growth and survival under extremely shaded conditions. 

The carbon starvation hypothesis is among the current leading hypotheses explaining the mechanism of plant mortality under stress—although it has not been verified, owing to a lack of available evidence [63]. Under 10% full sunlight, C starvation may occur when NSC cannot meet the requirements of the growth of seedling [67]. This may be explained by the fast-dying seedlings (*U. elongata*) having prioritized growth by consuming more NSCs for tissue growth, resulting in reduced metabolism and defense and then yielding an increase in *U. elongata* seedling mortality after deep shade treatment (e.g., 10% full sunlight) [63]. However, we did not determine when carbon starvation occurs in *U. elongata* seedlings under 10% full sunlight, and the threshold NSC and NSC content for carbon starvation in *U. elongata* should therefore be studied in future research. The SSRs reflect the nutrient utilization strategy of plants [45]. The SSRs of *U. elongata* seedling leaf, stem, and root tissues in response to light intensity levels of were studied. Overall, our results highlight their respective sensitivity, adaptive response under different light levels, and their most favorable light condition. Soluble sugar was preferred by the roots. The roots began to utilize starch under 40% and 10% full sunlight, indicating that the roots shift the carbohydrate use from growth to maintenance induced by the shade treatment in order to balance carbohydrate accumulation and plant growth. This type of trade-off between growth and defense benefits plants can create optimal living conditions under shade [68].

## 5. Conclusions

Changes in light intensity levels were observed to markedly affect the photosynthesis, nitrogen allocation, NSC allocation pattern, and C:N:P stoichiometry of *U*. *elongata* seedlings. Accordingly, 100% full sunlight was the most suitable light condition for seedling growth of *U. elongata*. More specifically, the seedlings grown under high light had higher *P*_n_, PNUE, *P*_C_, *P*_R_, *P*_B_, and NSC content values relative to those grown under shade. Thus, *U. elongata* exhibited adaptive strategies to light stress. The species exhibited strong internal nutrient stability under 40% full sunlight. These results indicate that more than 40% full sunlight level might be the favorable light condition for the improvement of the efficiency of *U. elongata* in understory environments, which can promote the regeneration of *U. elongata* seedlings. 

## Figures and Tables

**Table 1:** Effects of different light intensity levels on stomatal limitation (*L*_s_), carboxylation efficiency (*CE*), and water use efficiency (WUE) in *Ulmus elongata* leaves. Data are presented as the mean ± SE. Different small letters indicate significant differences at 0.05 levels.

Treatment	*L*_s_	*CE* (mmol·m^−2^·s^−1^)	WUE (mmol·mol^−1^)
100%	0.086 ± 0.004 a	39.96 ± 2.13 a	2.38 ± 0.04 b
40%	0.075 ± 0.007 b	23.99 ± 1.18 b	2.60 ± 0.07 a
10%	0.083 ± 0.007 a	19.18 ± 0.86 c	1.45 ± 0.03 c

**Table 2:** Effects of different light intensity levels on the photosynthetic nitrogen use efficiency (PNUE) and fractions of leaf N allocated to RuBisCO (*P*_R_), bioenergetic (*P*_B_), light-harvesting (*P*_L_), and photosynthetic components (*P*_C_) in *Ulmus elongata* leaves. Data are presented as the mean ± SE. Different small letters indicate significant differences at 0.05 levels.

Treatment	PNUE (μmol·g^−1^·s^−1^)	*P*_R_ (g·g^−1^)	*P*_B_ (g·g^−1^)	*P*_L_ (g·g^−1^)	*P*_C_ (g·g^−1^)
100%	13.50 ± 1.385 a	0.285 ± 0.015 a	0.025 ± 0.001 a	0.041 ± 0.006 b	0.350 ± 0.019 a
40%	10.33 ± 0.542 ab	0.211 ± 0.019 b	0.018 ± 0.002 b	0.051 ± 0.008 ab	0.280 ± 0.028 b
10%	6.67 ± 1.167 b	0.142 ± 0.007 c	0.012 ± 0.001 c	0.071 ± 0.002 a	0.224 ± 0.007 b

**Table 3:** Carbon, nitrogen, and phosphorus contents in the leaf, stem, and root tissues of *Ulmus elongata* seedlings in response to light intensity levels. Data are presented as the mean ± SE. Different letters indicate significant difference at 0.05 levels.

	Treatment	C (mg·g^−1^)	N (mg·g^−1^)	P (mg·g^−1^)
Root	100%	200.76 ± 5.45 a	7.93 ± 0.79 a	2.15 ± 5.45 a
	40%	178.50 ± 14.37 ab	4.14 ± 0.22 b	2.53 ± 0.30 a
	10%	135.94 ± 15.00 b	7.44 ± 1.12 a	1.21 ± 0.16 b
Stem	100%	202.85 ± 6.27 b	4.28 ± 0.17 b	2.18 ± 0.07 b
	40%	225.19 ± 8.05 b	5.32 ± 0.23 b	2.06 ± 0.07 b
	10%	278.93 ± 14.10 a	7.25 ± 0.44 a	2.79 ± 0.14 a
Leaf	100%	468.44 ± 7.68 a	14.15 ± 0.84 c	1.49 ± 0.02 a
	40%	412.91 ± 52.66 a	20.85 ± 0.47 b	0.98 ± 0.04 b
	10%	284.17 ± 11.39 b	25.76 ± 0.30 a	0.84 ± 0.01 c

**Table 4:** Effects of different light intensity levels on the C, N, and P stoichiometry of *Ulmus elongata* seedlings. Data are presented as the mean ± SE. Different letters indicate a significant difference at 0.05 levels.

	Treatment	C:N	C:P	N:P
Root	100%	25.76 ± 2.32 b	96.10 ± 10.50 b	3.79 ± 0.50 b
	40%	42.97 ± 1.35 a	71.94 ± 7.22 c	1.67 ± 0.15 c
	10%	18.57 ± 1.20 b	113.19 ± 4.61 a	6.16 ± 0.55 a
Stem	100%	47.48 ± 2.27 a	92.98 ± 5.33 b	1.97 ± 0.14 b
	40%	42.53 ± 2.65 ab	109.18 ± 10.41 a	2.58 ± 0.18 a
	10%	39.54 ± 0.55 b	100.11 ± 7.65 ab	2.60 ± 0.11 a
Leaf	100%	33.39 ± 2.36 a	313.73 ± 1.57 b	9.49 ± 0.67 c
	40%	19.76 ± 2.27 b	417.04 ± 36.41 a	21.27 ± 0.88 b
	10%	11.04 ± 0.47 c	338.61 ± 7.91 b	30.73 ± 0.60 a

## Data Availability

The data presented in this study are available from the corresponding author upon request.

## Supplementary Materials

The following supporting information can be downloaded at: https://www.mdpi.com/article/10.3390/life12091310/s1.

## Abbreviations

*P*_n_	Photosynthetic rate
*G*_s_	Stomatal conductance
*C*_i_	Intercellular CO_2_ concentration
*T*_r_	Transpiration rate
*CE*	Carboxylation efficiency
*L*_s_	Value of stomatal limitation
WUE	Water use efficiency
A	Leaf area
*N*_mass_	Leaf N per unit mass
*N*_area_	Leaf N per unit area
PNUE	Photosynthetic nitrogen use efficiency
*N*_mass_	Leaf N content per unit of leaf dry mass
*M*_A_	Leaf dry mass per unit area
*C*_Chl_	Leaf chlorophyll content
*V*_cr_	Specific activity of RuBisCO
*J*_mc_	Potential rate of photosynthetic electron transport per unit cytochrome f
*C*_B_	Ratio of leaf chlorophyll to leaf nitrogen invested in light harvesting
*P*_C_	Fractions of leaf N allocated to photosynthetic
*P*_R_	Fractions of leaf N allocated to RuBisCO
*P*_B_	Fractions of leaf N allocated to bioenergetics
*P*_L_	Fractions of leaf N allocated to light-harvesting
NSC	Non-structural carbohydrate
SSRs	The ratio of soluble sugar to starch
