# Supplementary material for: Photosynthesis, Nitrogen Allocation, Non-Structural Carbohydrate Allocation, and C:N:P Stoichiometry of Ulmus elongata Seedlings Exposed to Different Light Intensities
Source: Life (Basel). 2022 Aug 25;12(9):1310. doi: 10.3390/life12091310 (PMC9506466; doi:10.3390/life12091310)
Supplement: Supplementary file 1 [file life-12-01310-s001.zip › life-1851168-supplementary.pdf]

Table S1. The basic physical and chemical characteristics of soils. Data are presented as the mean ± SE.

Organic matter ($mg \cdot g^{-1}$)	Total nitrogen ($mg \cdot g^{-1}$)	Total potassium ($mg \cdot g^{-1}$)	Available potassium ($mg \cdot g^{-1}$)	Total phosphorus ($mg \cdot g^{-1}$)	pH
106.3±0.35	2.84±0.07	8.40±0.52	1.16±0.01	0.48±0.006	6.72±0.03

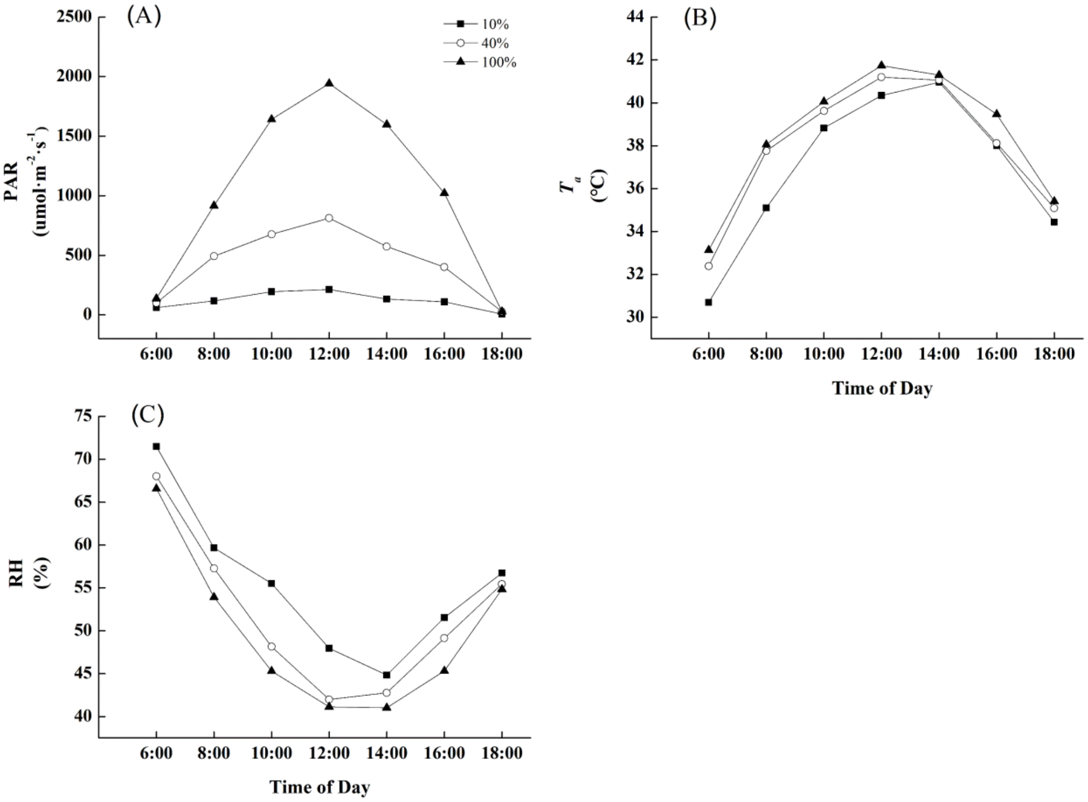

Figure S1. Diurnal variation in environmental factors under 100%, 40%, and 10% full sunlight, respectively. (**A**) Photosynthetically active radiation (PAR); (**B**) air temperature (T_a); (**C**) relative humidity (RH).

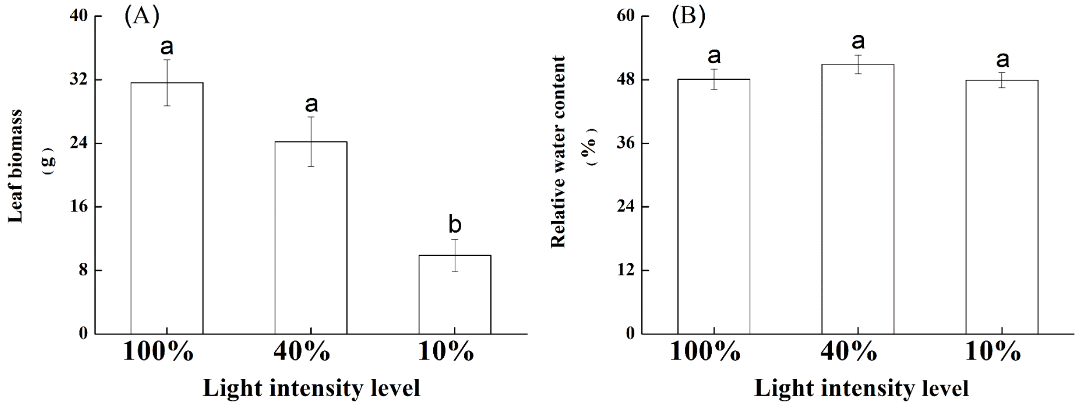

Figure S2. The leaf biomass (A), and leaf relative water content (B) in *U. elongata* seedlings under different light intensities. Data are presented as the mean ± SE. Different letters indicate significant difference at 0.05 levels.
